# Acute decompensated heart failure after transcatheter aortic valve implantation: A case report

**DOI:** 10.1002/ccr3.7597

**Published:** 2023-07-21

**Authors:** Hong Nyun Kim, Dong Heon Yang, Bo Eun Park

**Affiliations:** ^1^ Division of Cardiology, Department of Internal Medicine Kyungpook National University Hospital Daegu Korea; ^2^ Division of Cardiology, Department of Internal Medicine Kyungpook National University Chilgok Hospital Daegu Korea; ^3^ Department of Internal Medicine, School of Medicine Kyungpook National University Daegu Korea

**Keywords:** acute decompensated heart failure, left ventricular stunning, stress‐induced cardiomyopathy, suicide left ventricle, transcatheter aortic valve implantation

## Abstract

Transcatheter aortic valve implantation (TVAI) is a widely used treatment modality for severe aortic stenosis. The complication rates of the procedure have gradually decreased over time, owing to the improvements in procedural skills and development of TVAI devices. However, several rare but serious complications can still occur after TAVI. We recently encountered acute decompensated heart failure as a rare and fatal complication of TAVI and would like to share our experience.

## 1 INTRODUCTION

Since the first transcatheter aortic valve implantation (TAVI) was performed two decades ago, the TAVI procedure has undergone numerous advances. Currently, it is firmly established as the treatment of choice for severe aortic stenosis (AS) in patients with a high risk of surgical aortic valve replacement (SAVR).^1^ Although TAVI is less invasive than SAVR, it can also cause several periprocedural complications, including cerebrovascular accidents, ventricular perforation, valvular complications, arrhythmias, coronary artery occlusion, myocardial infarction, cardiogenic shock, and death.^2^ We recently encountered a case of acute decompensated heart failure (ADHF) after TAVI and report the details of the case herein.

## 2 CASE DESCRIPTION

**FIGURE 1:**
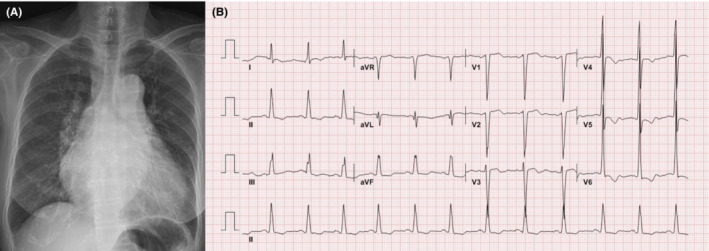
On the day of admission, Chest X‐ray (A) revealed cardiomegaly, but pulmonary edema and pleural effusion were not observed. Initial electrocardiogram (B) showed sinus rhythm and left ventricular hypertrophy with QRS widening.

**FIGURE 2:**
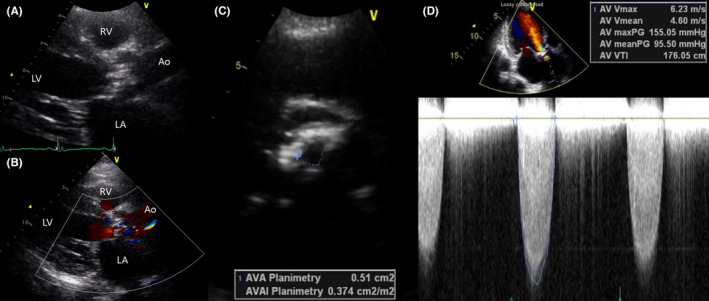
Initial transthoracic echocardiography (TTE) findings. (A, B) TTE showed left ventricular hypertrophy and degenerative aortic valve with severe calcification. (C) The aortic valve area (AVA) was 0.51 cm^2^ on the planimetry. (D) The mean systolic pressure gradient of aortic valve was 95.50 mmHg, and peak aortic jet velocity was 6.23 m/s on the continuous wave Doppler.

A 76‐year‐old female was transferred to our institution's emergency room due to exertional chest pain and dyspnea for a week. Two days prior to being transferred to our hospital, the patient visited a local hospital because of the aforementioned symptoms and subsequently underwent coronary angiography (CAG) and transthoracic echocardiography (TTE). CAG found no significant luminal stenosis, coronary plaque, or calcification in the coronary arteries; however, TTE showed severe AS. The patient had no prior medical history and was not taking any medications. The initial vital signs taken at the emergency room were as follows: blood pressure, 123/72 mmHg; heart rate, 78 bpm; respiratory rate, 18 breaths/min; and peripheral O_2_ saturation, 100% on room air. Cardiomegaly was observed on chest radiography, but no pulmonary edema or pleural effusion was noted (Figure 1A). An initial electrocardiogram (ECG) indicated sinus rhythm with a heart rate of 73 beats/min and a left ventricular (LV) hypertrophy pattern (Figure 1B). Laboratory examination did not indicate any abnormal findings, except for a slight increase in N‐terminal pro‐B‐type natriuretic peptide (NT‐proBNP) levels to 5618.0 pg/mL (reference range < 665.0 pg/mL) and high‐sensitivity cardiac troponin I (hs‐cTnI) to 0.552 ng/mL (reference range <0.034 ng/mL). TTE revealed very severe AS and mild decrease of left ventricular ejection fraction (LVEF) with global hypokinesia, as indicated by a LVEF of 40% by Simpson's method, aortic valve area (AVA) of 0.51 cm^2^ on planimetry, indexed AVA of 0.10 cm^2^/m^2^, mean pressure gradient of 95.50 mmHg, and peak aortic jet velocity of 6.23 m/s (Figure 2). The patient's perioperative mortality rate was evaluated, and subsequently, she was classified into the intermediate‐risk group with a Society of Thoracic Surgeons risk score of 6.806%. We discussed the treatment options (SAVR vs. TAVI) with the patient in the presence of a multidisciplinary team of interventional cardiologists, cardiac surgeons, and anesthesiologists until the decision to perform TAVI was reached. Prior to TAVI, a computerized tomography scan was performed to evaluate the type and size of the prosthetic valve required and peripheral vascular approach needed.

**FIGURE 3:**
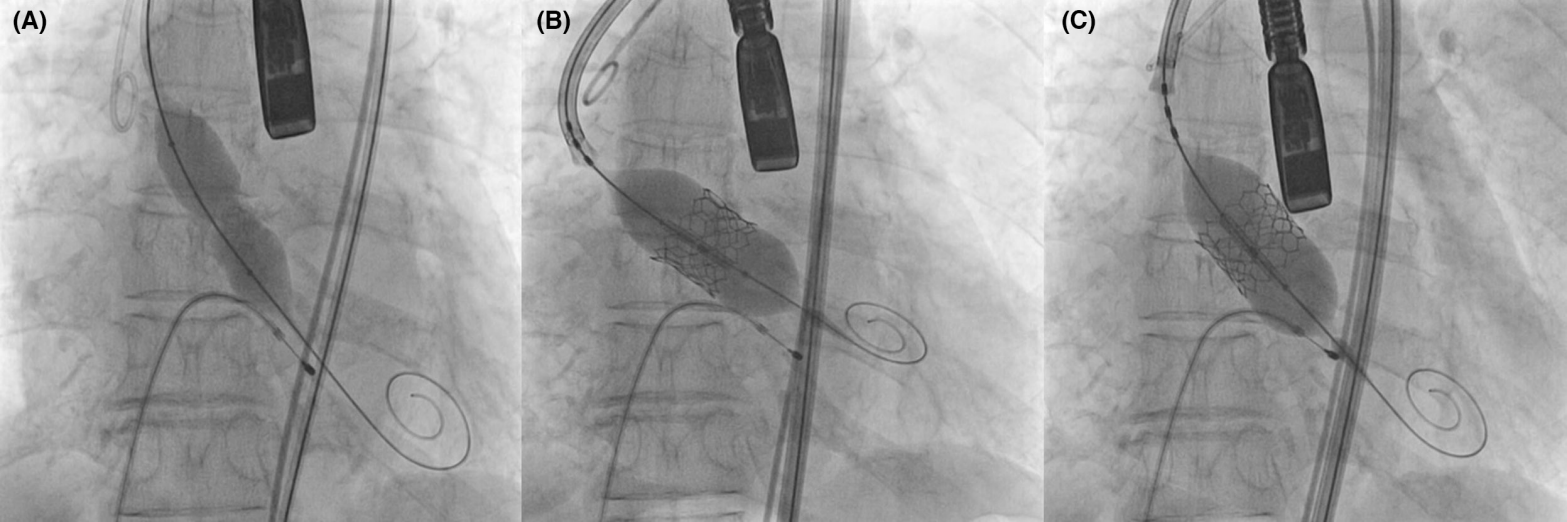
Transcatheter aortic valve implantation (TAVI) procedure. (A) Pre‐balloon dilation was performed under rapid ventricular pacing (RVP). (B) An Edwards SAPIEN 3, 23 mm, prosthetic aortic valve was successfully implanted. (C) Post‐balloon dilation was performed under RVP to reduce paravalvular leakage.

**FIGURE 4:**
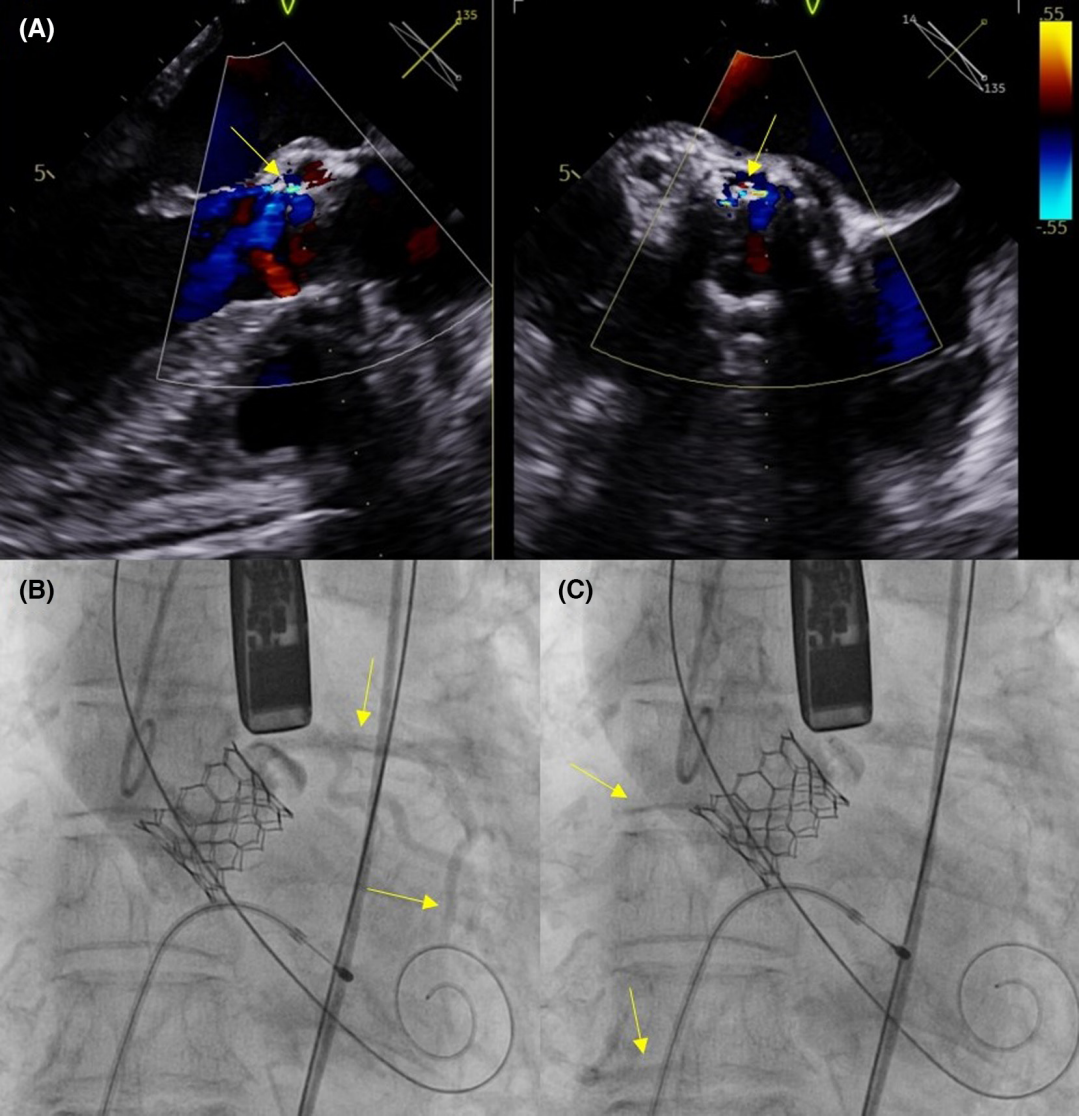
Post TAVI procedural findings (A) Transesophageal echocardiography showed minimal paravalvular leakage (arrow). (B, C) Coronary artery blood flow was intact on aortography after the TAVI procedure.

The TAVI procedure was performed 2 days after hospitalization under general anesthesia via the right femoral artery. An Edwards SAPIEN 3 prosthetic aortic valve (23 mm; Edwards Lifesciences) was implanted with no specific events or complications occurred during the procedure (Figure 3). However, in this particular case, four rapid ventricular pacings (RVP) were conducted at a pacing rate of 160 bpm to achieve appropriate implantation depth and position, which is typically performed only once. After the procedure, minimal paravalvular leakage (PVL) and trace aortic regurgitation (AR) were observed on transesophageal echocardiography, and the implanted aortic valve functioned well (Figure 4). Coronary blood flow was intact on aortography after the procedure (Figure 4). Finally, the patient's vital signs were stable with a blood pressure 128/66 mmHg and a heart rate of 76 bpm, and the procedure concluded without acute complications. After the procedure, the patient was admitted to the intensive care unit for observation. However, 1 h after the procedure, the patient's blood dropped to 42/25 mmHg. Intravenous hydration was promptly initiated with normal saline, and inotropic support with norepinephrine was administered to restore the patient's vital signs. Subsequently, the patient's blood pressure stabilized at 130/67 mmHg and heart rate at 83 bpm. We examined the peripheral procedure site, but no specific complications such as bleeding and hematoma were observed. Additionally, there were no difference found in the ECG compared to the previous examination. A complete blood count, which included hemoglobin (13.4 g/dL) and hematocrit (39.9%) showed normal values. However, an arterial blood gas study revealed the presence of metabolic acidosis (pH 7.253; PCO_2_, 41.0 mmHg; HCO3, 16.6 mmol; base excess, −9.1 mEq). Additionally, the patient's cardiac enzyme, hs‐cTnI, was 1.402 ng/mL, indicating a modest increase compare to the initial value. On TTE, the implanted aortic valve was observed to be functioning well, and there were no significant changes in the amount of AR and PVL observed, which were similar to the last images taken. No significant abnormal findings were observed in the other valves, and there was no evidence of pericardial effusion. Despite these results, the patient's systolic heart function continued to deteriorate, with a LVEF of 25% and global hypokinesia. Furthermore, the patient's blood pressure continued to decrease despite the administration of inotropes such as epinephrine and vasopressin, the lactic acid levels increased to 17 mmol/L. At 7 h post‐TAVI, mechanical ventilation, continuous renal replacement therapy, and venoarterial extracorporeal membrane oxygenation (VA‐ECMO) were initiated in an effort to improve patient's hemodynamics. Despite the continuation of supportive care with mechanical support, the patient's heart function continued to deteriorate and ultimately fail to recover. The patient eventually died, 3 days after the TAVI procedure.

## 3 DISCUSSION

Transcatheter aortic valve implantation has revolutionized the treatment of severe AS since its inception in 2002. Initially indicated for inoperable patients or those deemed too high risk for SAVR, the scope of its application gradually expanded due to technological advances and various successful study results.^1^ Despite its advanced technique and widespread use, TAVI can result in several life‐threatening complications, including coronary obstruction, ventricular rupture, vascular injury, stroke, and death.^2^ ADHF may also occur after TAVI from numerous causes, including LV dysfunction (due to coronary obstruction, “stunning” via rapid pacing, or stress‐induced cardiomyopathy), arrhythmia, annular rupture, pericardial effusion, mitral valve insufficiency, peripheral vascular injury, and a “suicide ventricle” (hyperdynamic intraventricular obstruction after unloading by TAVI).^2^, ^3^, ^4^, ^5^

In the present case, the possibility of coronary obstruction due to an implanted aortic valve was considered low, as confirmed by an aortogram post‐TAVI that showed patency of the coronary artery. During the progression of cardiogenic shock, continuous ECG monitoring did not detect any ST segment changes or ventricular arrhythmias, such as ventricular fibrillation or ventricular tachycardia. Additionally, the patient remained alert and did not complain of chest pain or discomfort. TTE was performed repeatedly, but apart from a severe decrease in LVEF with global hypokinesia, no abnormal findings were detected that could cause cardiogenic shock, such as insufficiency of implanted aortic valve, mitral valve insufficiency, pericardial effusion, ventricular septal defect, and left ventricular outflow tract (LVOT) obstruction. No complications, such as bleeding or hematoma, were observed in the peripheral vessels used during the TAVI procedure. Furthermore, the patient's red blood cell count and hematocrit levels were within the normal ranges. Continuous ECG monitoring was performed after the procedure, and no conduction abnormalities, such as bradycardia or atrioventricular block, were detected. Although there were few clinical indications of acute coronary artery obstruction in this patient, we were unable to perform coronary angiography to definitively rule out the possibility when the patient's condition worsened. This limitation is a major drawback of this report.

Except for acute coronary obstruction, three possible causes of ADHF were considered in this patient. First, it is conceivable that the patient experienced post‐procedural stress‐induced cardiomyopathy(SCMP) accompanied by heart failure with mid‐range ejection fraction (HFmrEF). SCMP is a clinical syndrome characterized by acute and transient (<21 days) LV systolic and diastolic dysfunction often related to an emotional or physical stressful event.^6^ Clinically, SCMP is recognized to exhibit a benign course with an in‐hospital mortality rate of 1%–2%, although some meta‐analyses report higher rates of 3.5%–4.5%.^7^ Additionally, a study of patients with high severity of illness reported up to 16% mortality during hospitalization, indicating a poor prognosis when the severity of underlying disease is high or when SCMP perpetuates a vicious cycle in the course of the disease.^8^ Systolic HF is the most frequently encountered complication during the acute phase of stress‐induced cardiomyopathy, with a reported incidence of 12%–45% of cases. For mild cases, observation or a short‐term use of pharmacological therapy may be considered. However, in severe cases that are complicated by progressive circulatory failure, mechanical circulatory support may be required as a bridging strategy for selected patients.^6^ Although SCMP related to the TAVI procedure is very rare, a few cases have been reported in the literature.^4^, ^5^ In this particular case, we performed four RVPs for pre‐balloon dilation, prosthetic valve implantation, and post‐balloon dilation. Each RVP induces a drop in blood pressure that can lead to SCMP. Furthermore, both pre‐ and post‐balloon dilation can also induce SCMP. Second, transient ventricular stunning due to RVP and balloon aortic valvuloplasty (BAV) could have caused ADHF in the patient. Ventricular stunning is an established clinical entity that has already been described in TAVI.^3^, ^9^, ^10^, ^11^ Despite the immediate relief of the LVOT obstruction, TAVI patients experience myocardial injury from an ischemic‐reperfusion injury due to rapid pacing.^12^ The etiology of global myocardial stunning after rapid pacing involves the creation of free radical species and intracellular calcium over‐load.^13^ Both RVP and BAV induce short periods of myocardial ischemia that can precipitate cardiogenic collapse due to profound ventricular stunning in susceptible patients. Patients with coronary artery disease, significantly reduced oxygen levels during RVP, or marked LV hypertrophy, which can increase oxygen demand, are at risk of myocardial ischemia during RVP.^3^ Generally, patients undergoing TAVI are older adults with coronary artery stenosis and severe AS, often presenting with LV hypertrophy, and hypertrophied ventricles may be more susceptible to myocardial damage, in part due to the reduced myocardial capillary density and higher intracavitary pressure.^12^ Therefore, the possibility of ventricular sunning due to RVP is higher in this group compared to the general population. In this case, the patient had LV hypertrophy and underwent four RVPs and three balloon inflations during the TAVI procedure, which could have triggered ventricular stunning. Finally, it is important to note that suicide of the left ventricle can cause cardiogenic shock without coronary obstruction. Rapid changes in LV function may occur after the TAVI procedure, leading to an increase in dynamic intraventricular gradients (DIG). These changes can cause dynamic LVOT obstruction with systolic anterior motion of the mitral valve (SAM), resulting in a phenomenon known as “suicide left ventricle”. This phenomenon has been reported in both SAVR and TAVI procedures.^14^, ^15^ An echocardiographic study was conducted to determine predictive factors for the development of DIG following aortic valve replacement.^16^ The study found that small LV end‐diastolic diameter, high ejection fractions, high interventricular septum to posterior wall thickness ratios, high valve gradients, and small LV masses were identified as predictive factors for DIG.^16^ In our patient, preoperative TTE revealed an LV end‐diastolic diameter of 5.5 cm, and interventricular septum and posterior wall thickness of 11 mm each, with an LVEF of 40%. Furthermore, the TTE performed during the episode of cardiogenic shock showed no evidence of SAM or LVOT obstruction, but did reveal severe LV dysfunction. Based on these findings, it is unlikely that the patient was experiencing suicide left ventricle.

Heart failure is known to be one of the leading causes of death following the TAVI procedure.^17^ The main cause of heart failure is typically myocardial ischemia due to acute coronary obstruction. However, in addition to acute coronary obstruction, SCMP or LV stunning also have the potential to cause ADHF. Proper preparation for serious but rare complications such as SCMP and LV stunning is important when planning and performing TAVI. Certain factors, such as being elderly female, having low body weight, hypertrophied ventricles, discontinuing beta‐blockers, and having coronary artery disease, have been identified as risk factors for developing SCMP and LV stunning.^12^

## 4 CONCLUSION

To reduce the risk of these complications in high‐risk patients, strategies such as reducing the number and rate of rapid ventricular pacing, avoiding pre‐balloon dilation, using a smaller balloon size for pre‐balloon dilation, and implanting a self‐expandable prosthetic aortic valve should be considered. Careful consideration of these factors and appropriate management of high‐risk patients can help reduce the risk of serious complications following TAVI.

## AUTHOR CONTRIBUTIONS

**Hong Nyun Kim:** Conceptualization; data curation; investigation; writing – original draft; writing – review and editing. **Dong Heon Yang:** Conceptualization; supervision. **Bo Eun Park:** Writing – review and editing.

## FUNDING INFORMATION

None.

## CONFLICT OF INTEREST STATEMENT

All authors have no potential conflicts of interest to disclose.

## ETHICS STATEMENT

The study protocol was reviewed and approved by the Institutional Review Board of the School of Medicine, Kyungpook National University (IRB No. 2022‐09‐021), Daegu, Korea.

## CONSENT

Written informed consent was obtained from the patient to publish this report in accordance with the journal's patient consent policy.

## Data Availability

All data generated or analyzed during this study are included in this published article.
